# Measuring Resilience and Fatality Rate During the First Wave of COVID-19 Pandemic in Northern Italy: A Note

**DOI:** 10.3389/phrs.2022.1604308

**Published:** 2022-06-20

**Authors:** Calogero Guccio

**Affiliations:** ^1^ Department of Economics and Business, University of Catania, Catania, Italy; ^2^ Health Econometrics and Data Group, University of York, Heslington, United Kingdom

**Keywords:** resilience, biased indicator, missing data, epidemiology, COVID-19, Healthcare governance, Italy

## Abstract

**Background:** This Policy Brief aims to contribute to the debate on the resilience of the healthcare systems during the pandemic by discussing whether mortality indicators are appropriate for assessing resilience or whether other statistics should be employed.

**Evidence:** During the first wave of the COVID-19, much emphasis was placed on case-fatality rates to offer a preliminary assessment of the resilience of healthcare systems. However, these statistics are often biased and do not consider the real figure of the population that has been infected.

**Policy Options and Recommendations:** Comparing data obtained with different approaches based on statistical inference and large-scale serological survey, the brief highlights, that great care must be taken when using case-fatality data, which in the absence of careful analysis, can lead to erroneous conclusions.

**Conclusion:** Using case-fatality rate gives us no sounding information about the real capability of healthcare systems to save lives during the pandemic. However, even in the absence of detailed epidemiological data new advancements in statistical methods can be useful to provide a more sounding evaluation of the resilience of the healthcare systems.

## Background

The resilience of healthcare systems to exogenous shocks is an issue that has taken on dramatic importance during the COVID-19 pandemic. Compared with other fields, the concept of the resilience of healthcare systems is relatively recent [1] and even more recent is the still very limited attempt to identify measures of resilience in the healthcare field [2]. Nonetheless, it seems quite reasonable to assume that this concept has several dimensions [3, 4].

During the first assessments of the pandemic, much emphasis was placed on mortality indicators such as case-fatality rate (i.e., the number of deaths in persons who tested positive for infection divided by the number of tested positive cases), or crude mortality data (i.e., the number of deaths divided by the population) and standardized by population mortality rates (i.e., the number of deaths divided by the population adjusted to a standard age distribution) to propose comparisons between healthcare systems [5]. Although it has been observed that several biases render the case-fatality rates of little epidemiological value [6] some contributions in the literature have proposed this index to assess the resilience of healthcare systems.

In this perspective, a recent paper by Costa Font et al. [7] takes the opportunity offered by the pandemic to evaluate the resilience of three Italian regional healthcare systems characterized by different management competition (MC) models. The topic is of crucial importance not only to provide answers on what happened in Italy during the first wave of the pandemic crisis but above all to draw lessons for the future.

The main thesis of the authors is that highly decentralized healthcare systems like Lombardy have shown during the first wave of the COVID-19 pandemic less resilience than centralized ones. In particular, the authors observe that the lack of incentive to cooperate, the higher transaction costs and the absence of coordination in Lombardy resulted in more fatalities compared with the more centralized regional healthcare systems of Emilia Romagna and Veneto. To provide empirical support to their thesis the authors report, for the three regions, comparative data on hospital ownership, COVID-19 mortality rates and swabs per capita.

On this matter, Connelly and Birch [8] notice that more detailed epidemiological data than those currently available are needed to provide a sound answer to this key question. Along the same lines of reasoning, Bel and Esteve [9] make a comparison of mortality and morbidity between OECD (Organization for Economic Co-operation and Development—OECD is an international organization based in Paris and brings together 38 developed countries that share a market economy) countries with different health management systems and between regions in Spain with a different degree of healthcare decentralization and they do not find any link between MC models and the severity of fatalities.

However, both analyses are not conclusive to assess the resilience of healthcare systems because the case-fatality rate and crude mortality data are not only likely to be biased but, more importantly, do not reflect the real figure of the population that has been infected with COVID-19. Furthermore, while we know how many people have received a positive test result, most have not been tested, and tests do not have perfect accuracy. Hence, using the crude case-fatality rate for COVID-19 gives us no information about the capability of healthcare systems to save lives during the pandemic. Additionally, the use of population-standardized mortality rates for COVID-19 to assess the resilience may lead to incorrect conclusions if the count of death is imprecise [10, 11] and the spread of infection is largely different between healthcare systems [12]. To assess the resilience in the pandemic context, in our view, the most reasonable mortality measures to adopt are those of variation in avoidable all-cause mortality after the exogenous shock and the infection fatality rate (i.e., the proportion of the infected population that has died). However, the first indicator requires complex epidemiological analyses that, to the best of our knowledge, are largely not yet available. Thus, for convenience, in this contribution, we focus only on the latter.

Finally, the correct inference of the real number of infections is crucial to our understanding of the public health impact of the COVID-19 pandemic and on the resilience of different healthcare systems.

In this perspective, a large body of authoritative literature has now definitively clarified that the problem of undetected cases in the first wave of the pandemic did not only concern asymptomatic or paucisymptomatic individuals that in principle do not require large treatment and therefore have a limited impact on the resilience of the healthcare system (e.g., [10, 12]).

Moreover, asymptomatic and paucisymptomatic individuals also require public health policies and resources to prevent them from spreading the infection. Indeed, in the absence of correct information on the prevalence of infected patients—even if they are asymptomatic—public health policies to fight the pandemic are ineffective [13]. In this regard, effective surveillance health policies originated by the correct inference of infection numbers certainly have a relevant impact on the resilience of the healthcare system.

The rest of this brief is structured as follows. *Evidence* section outlines that the use of crude mortality indicators to provide a preliminary assessment of the resilience of health systems during the first pandemic wave may lead to biased assessments. *Policy Options and Recommendations* section, comparing data from different approaches, highlights that, recent statistical methods can provide a more robust assessment even in the early stages of pandemics and recommends their use. *Conclusion* section summarizes the main conclusions.

## Evidence

Given the weaknesses of indicators such as the case-fatality rate, a more appropriate indicator would be the infection fatality rate (i.e., the proportion of the infected population that has died). The infection fatality rate, unlike the case-fatality rate, considers all infected individuals who have died and not only those who have been identified.

In the first wave of the pandemic the initially inadequate number of testing kits, testing quality and facilities determined that the number of confirmed cases as a proportion of the total population underestimated the spread of infection rate in several countries [12]. It follows that the case fatality rate was particularly biased at both the numerator (i.e., individuals who died from COVID-19) and the denominator (i.e., individuals infected with COVID-19). Individuals who died of COVID-19 but were not tested were not included in the numerator of the case fatality rate: as a result, the number of deaths from COVID-19 underestimates true deaths from the infection [10]. Similarly, asymptomatic, and mildly symptomatic infections largely were not tested - greatly underestimating the true number - were not considered in the denominator [12, 13].

Estimating the infection fatality rate facilitates the identification of vulnerable segments of the population and informs key policy decisions to mitigate the consequences of the pandemic. Unfortunately, it cannot be calculated without an accurate count of infections within the population, including asymptomatic cases. Nevertheless, several epidemiological and statistical approaches have been proposed to assess the real spread of COVID-19 infection [14].

Among others, a recent paper by [15] has proposed a sound statistical method with minimal assumptions for using currently available administrative information to better understand cumulative infection rates and infection fatality rates. Other empirical approaches include the study by [16], which uses travel patterns to estimate the number of unreported infections that can be used to calculate a plausible infection mortality rate. However, the approach in [16] requires stronger assumptions and more data than [15].

Manski and Molinari [15] apply their partial identification analysis to available administrative data (i.e., data collected by the healthcare system) from Illinois, New York, and Italy to generate upper and lower bounds on the rates of COVID-19 infection. For the case of Italy on 24th April 2020, the authors estimate wide bounds of plausible infection rate ranging from 0.6% to 47.1% of the population. However, even with these wide bounds, the estimates contain useful information to assess the plausible cumulative infection fatality rate in Italy. For instance, whereas as of 24th April in Italy, 13.4% of official confirmed cases resulted in death, the estimated bounds on infection rates on the same date imply a significantly lower infection fatality rate, ranging from 0.1% to 7.7% of infected individuals. Moreover, the authors report that, if the evidence from the town of Vò in Italy [17], is applied to the estimated model for the whole country, the upper bound must be multiplied by 0.568 and the updated bounds of the more plausible cumulative infection fatality rate in Italy range at the same date from 0.1% to 4.4% of infected individuals. The reader interested in the mathematical details of epidemiological and inference models to assess the real spread of COVID-19 infection can refer to [15, 18, 19].

In the absence of more detailed epidemiological data, this work demonstrates what can be learned about cumulative infection rates and infection fatality rates from administrative information that is more readily available. Additionally, these estimates are substantially different from the mortality rates observed in the patients that have received a positive test result. This marked difference between case-fatality rates and infection fatality rates is largely due to the number of untested infected population, which in the first wave of the pandemic in Italy was very large. Thus, to provide a first statistical sound testing of the thesis of [7], it seems important to carry out further investigations to assess the plausible infection fatality rate and the resulting performance in the three Italian regions.

## Policy Options and Recommendations

A different approach to evaluate the true infection rate in the population is serological surveys of antibodies for COVID-19 [20]. Serological surveys for an appropriately stratified random sample of the population provide an estimate of the number of people in the population who have antibodies to the virus at a given point in time, providing a robust estimate of the level and trend of infection rate [20].

In this respect, the preliminary data of a recent large-scale serological survey conducted by the Italian Statistical Office (ISTAT) between 25th May and 15th July 2020, revealed that around 1.5 million Italians were infected at that date with a cumulative infection rate of 2.5% at country level [21].

**FIGURE 1:**
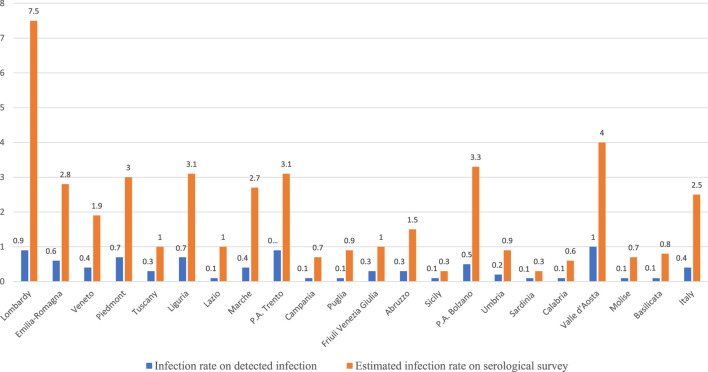
Cumulative infection rate at the regional level (Italy, 2020). Source: author’s computation on data provided by Istituto Superiore di Sanità, (https://github.com/pcm-dpc/COVID-19/blob/master/schede-riepilogative/regioni/dpc-covid19-ita-scheda-regioni-20200715.pdf) and Italian Statistical Office (ISTAT) and Ministero della Salute (http://www.salute.gov.it/imgs/C_17_notizie_4998_0_file.pdf).

The study also shows a significant difference between Italian regions in terms of the cumulative infection rate [22]. Estimates in Figure 1 show a cumulative infection rate of 7.5% in Lombardy compared to 1.9% in Veneto and 2.8% in Emilia Romagna, and very low infection rate figures in southern Italy. These statistics are significantly different from those obtained through administrative data also shown in Figure 1. The cumulative infection rate computed on administrative data was 0.9 in Lombardy, 0.6 in Emilia Romagna and 0.4 in Veneto, respectively. Thus, the reasonably real figures in the population that have been infected with COVID in Lombardy on the date of 15th July 2020 were eight times larger than that detected through the swabs.

These data can provide a first basis for assessing more accurately the performance of Italian regional systems against the first wave of the pandemic by calculating a plausible real infection fatality rate and thus a better approximation of the healthcare performance to save the lives of people affected by the infection. Table 1 reports the data, on 15th July 2020, of the cumulative infection fatality rate using administrative data and data from the serological survey. The first part of Table 1, which uses administrative data, describes a picture that overlaps with that of Costa Font et al. [7]. The infection fatality rate and standardized mortality rates in Lombardy were much higher than those in Emilia Romagna and Veneto. However, if we use data from the serological survey to calculate the infection rate, the assessment is completely reversed. The plausible infection fatality rate for Lombardy was 2.23, virtually indistinguishable from that of Veneto (2.20) and much better than that of Emilia Romagna (3.42). Interestingly enough, the infection fatality rate at the national level of 2.35 reported in Table 1 is largely in line with those obtained by [15]. Thus, if we look at the data on the plausible spread of the virus, we cannot say that the healthcare system in Lombardy did a bad job compared with Veneto and Emilia Romagna.

**TABLE 1:** Computation of cumulative infection fatality rate (Italy, 2020).

Regions	Population^a^	Computation using administrative data^b^					Computation using serological survey^c^		
		Cumulative infections	Cumulative dead	Infection rate on detected infection	Infection fatality rate	Standardized mortality per 100,000 inhabitants	Estimated infection rate	Estimated infected population	Estimated infection fatality rate
Lombardy	10,027,602	95,236	16,765	0.9	17.60	167.19	7.5	752,070	2.23
Emilia-Romagna	4,464,119	28,989	4,271	0.6	14.73	95.67	2.8	124,995	3.42
Veneto	4,879,133	19,441	2,043	0.4	10.51	41.87	1.9	92,704	2.20
Piedmont	4,311,217	31,515	4,118	0.7	13.07	95.52	3.0	129,337	3.18
Tuscany	3,692,555	10,338	1,127	0.3	10.90	30.52	1.0	36,926	3.05
Liguria	1,524,826	10,042	1,561	0.7	15.54	102.37	3.1	47,270	3.30
Lazio	5,755,700	8,376	847	0.1	10.11	14.72	1.0	57,557	1.47
Marche	1,512,672	6,805	987	0.4	14.50	65.25	2.7	40,842	2.42
P.A. Trento	545,425	4,881	405	0.9	8.30	74.25	3.1	16,908	2.40
Campania	5,712,143	4,787	432	0.1	9.02	7.56	0.7	39,985	1.08
Puglia	3,953,305	4,541	547	0.1	12.05	13.84	0.9	35,580	1.54
Friuli Venezia Giulia	1,206,216	3,339	345	0.3	10.33	28.60	1.0	12,062	2.86
Abruzzo	1,293,941	3,331	467	0.3	14.02	36.09	1.5	19,409	2.41
Sicily	4,875,290	3,115	283	0.1	9.09	5.80	0.3	14,626	1.93
P.A. Bolzano	532,644	2,677	292	0.5	10.91	54.82	3.3	17,577	1.66
Umbria	870,165	1,452	80	0.2	5.51	9.19	0.9	7,831	1.02
Sardinia	1,611,621	1,376	134	0.1	9.74	8.31	0.3	4,835	2.77
Calabria	1,894,110	1,218	97	0.1	7.96	5.12	0.6	11,365	0.85
Valle d'Aosta	125,034	1,196	146	1.0	12.21	116.77	4.0	5,001	2.92
Molise	300,516	446	23	0.1	5.16	7.65	0.7	2,104	1.09
Basilicata	553,254	405	27	0.1	6.67	4.88	0.8	4,426	0.61
Italy	59,641,488	243,506	34,997	0.4	14.37	58.68	2.5	1,491,037	2.35

a Italian Statistical Office (ISTAT), http://dati.istat.it/Index.aspx?DataSetCode=DCIS_POPRES1#.

b Data on 15th July 2020 from Istituto Superiore di Sanità, https://github.com/pcm-dpc/COVID-19/blob/master/schede-riepilogative/regioni/dpc-covid19-ita-scheda-regioni-20200715.pdf.

c Data on 15th July 2020 from Italian Statistical Office (ISTAT) and Ministero della Salute; http://www.salute.gov.it/imgs/C_17_notizie_4998_0_file.pdf.

Source: author’s computation from above mentioned source.

Previous figures highlight that it is probably true that structural and organizational weaknesses of regional healthcare systems may have contributed to some extent to the (mis)management of the pandemic in Italy. However, much effort still needs to be made to assess the actual impact of the specific organizational model on the resilience of a healthcare system to exogenous shocks such as pandemics. Although undoubtedly complex, such an effort must be undertaken to avoid repeating tragic errors in the future.

From a public health policy perspective, in this Section, by comparing data from different approaches, we have highlighted how, even in the absence of detailed epidemiological data, new advances in statistical methods can be useful in providing a more robust assessment of resilience of health systems. Moreover, these statistical methods are relatively easy to compute and allow for a better inference of the spread of infection even during the early stages. For these reasons, their use is recommended in the most recent literature [18, 19].

## Conclusion

This policy brief aimed to contribute to the debate on the resilience of the healthcare systems during the pandemic by discussing whether the case-fatality rate and crude mortality indicators are appropriate for assessing resilience or whether other indicators should be employed. Comparing data obtained with different approaches based on statistical inference and large-scale serological survey, the article underlined the weakness of crude mortality indicators. Furthermore, it showed that even in the absence of detailed epidemiological data new advancements in statistical methods can be useful to provide a more sounding evaluation of the resilience of the healthcare systems.

However, some caveats of our analysis must be discussed. The analyses and data we have reported here are certainly preliminary, and much work still needs to be done to fully understand the resilience of health systems to the pandemic. We believe, however, that great care must be taken when using case-fatality and crude mortality data, which in the absence of careful analysis can lead to erroneous conclusions.

Furthermore, if the performance of healthcare systems during a pandemic is measured by the capability to save the lives of infected people, our analysis shows that the healthcare system performance in Lombardy was not significantly different from those of other regions with a different MC model. However, it could be argued that the performance of healthcare systems during a pandemic should be measured in terms of their ability to contrast the spread of the pandemic among the population. In this case, the significant difference in the infection rate between Lombardy and the other Italian regions would tend to support the thesis of [7]. However, many studies show that the spread of infection in a geographical area depends on several factors, including behavioural factors (e.g. [23, 24]), and not only on the organization of the healthcare system. In this perspective, when more robust data become available, a more thorough analysis of the resilience of healthcare systems in Lombardy will be feasible by analysing the variation in avoidable all-cause mortality after the exogenous shock of COVID-19.

In conclusion, the use of crude mortality rates does not provide us with reliable information on the true ability of healthcare systems to save lives during a pandemic. However, even in the absence of detailed epidemiological data such as those derived from serological surveys, which, however, require relatively long timeframes and representative population samples, new advances in statistical methods may be useful in providing a more robust assessment of the resilience of health systems.
